# Switchable Glass Enabled Contextualization for a Cyber-Physical Safe and Interactive Spatial Augmented Reality PCBA Manufacturing Inspection System

**DOI:** 10.3390/s20154286

**Published:** 2020-07-31

**Authors:** Joel Murithi Runji, Chyi-Yeu Lin

**Affiliations:** 1 Department of Mechanical Engineering, National Taiwan University of Science and Technology, Taipei 106, Taiwan; d10403803@mail.ntust.edu.tw; 2 Center for Cyber-Physical System Innovation, National Taiwan University of Science and Technology, Taipei 106, Taiwan; 3 Taiwan Building Technology Center, National Taiwan University of Science and Technology, Taipei 106, Taiwan

**Keywords:** spatial augmented reality, automatic optical inspection, cooperation, printed circuit board assembly, switchable glass, smart manufacturing, projector, Industry 4.0, cyber-physical

## Abstract

Augmented reality (AR) has been demonstrated to improve efficiency by up to thrice the level of traditional methods. Specifically, the adoption of visual AR is performed widely using handheld and head-mount technologies. Despite spatial augmented reality (SAR) addressing several shortcomings of wearable AR, its potential is yet to be fully explored. To date, it enhances the cooperation of users with its wide field of view and supports hands-free mobile operation, yet it has remained a challenge to provide references without relying on restrictive static empty surfaces of the same object or nearby objects for projection. Towards this end, we propose a novel approach that contextualizes projected references in real-time and on demand, onto and through the surface across a wireless network. To demonstrate the effectiveness of the approach, we apply the method to the safe inspection of printed circuit board assembly (PCBA) wirelessly networked to a remote automatic optical inspection (AOI) system. A defect detected and localized by the AOI system is wirelessly remitted to the proposed remote inspection system for prompt guidance to the inspector by augmenting a rectangular bracket and a reference image. The rectangular bracket transmitted through the switchable glass aids defect localization over the PCBA, whereas the image is projected over the opaque cells of the switchable glass to provide reference to a user. The developed system is evaluated in a user study for its robustness, precision and performance. Results indicate that the resulting contextualization from variability in occlusion levels not only positively affect inspection performance but also supersedes the state of the art in user preference. Furthermore, it supports a variety of complex visualization needs including varied sizes, contrast, online or offline tracking, with a simple robust integration requiring no additional calibration for registration.

## 1. Introduction

Quality in light of customer satisfaction is a key ingredient towards sustainability and the productivity of manufacturing organizations. In this regard, inspection at various levels of production is inevitable from the acquisition of raw material components to assembly of final products. A key part in all electronic products is printed circuit board assemblies (PCBAs), that function as the controllers of these products and whose failure can be catastrophic. Its importance cannot be overstated herein yet the need for stable, exceptionally reliable, durable, high performance without defects, in compact form, and at low cost [1] would not be underrated either. This unique blend of requirements builds complexity arising from not only layering of the printed circuit boards (PCBs), to corresponding miniaturization of assembly electronic components, but also its susceptibility to defects emerging from high throughput to satisfy demand and customization requirements. These unique set of needs necessitates a review of existing double-check/inspection systems and proposes an efficient novel method that leverages modern technologies in line with Industry 4.0 precepts.

The inspection of PCBAs is regularly performed during and after manufacturing assembly to ensure proper parts are correctly localized on right boards [2]. This inspection is often undertaken through automatic optical inspection (AOI) machines [3] complemented by high-skill inspectors. The inspector is required to verify whether the defects previously identified and localized by an AOI system could affect the PCBA’s functionality upon product completion. Early error detection promotes savings in material and energy consumption. In turn, this would result in increased product quality, uniformity, reliability, productivity, customer satisfaction with a corresponding reduction in waste and rework required. To benefit appropriately from this defect information available from AOI systems that facilitates early and efficient defect detection, real-time, on demand, cyber-physical integration is necessary for precise visualization of defect regions on the PCBA. Accordingly, this ushers in augmented reality (AR) technology interfacing.

Augmented reality is a technology that combines real with interactive virtual components in real time, in situ and in the three-dimensional physical environment [4,5,6,7,8,9,10]. The precisely registered virtual components could be audio, visual or haptic type with an aim of modifying a user’s perception [7]. We shall focus on visual components in this study. Visualization, of virtual components can be assisted by either large screens, tablets, smartphones, see-through head mounted devices or projectors. Employing large screens limits its scalability in different industrial settings while simultaneously experiencing a high angular offset [11]. Tablets and smartphones offer low cost visualization on one hand, yet hinder the maneuverability of operators on the other owing to their manual handling needs [11]. Head-mounted devices (HMDs) alternatively need to be worn by users [12,13], involve complex tracking [13], have limitations in field-of-view (FOV) [12,13], resolution [12], registration, are bulky [12,13], and have an high chance of causing simulator sickness by downgrading the user’s perception to the resolution, frame-rate and latency of the display [12]. Consequently, this draws us to projector-based visualization, that addresses most of these concerns yet is not fully explored.

Spatial augmented reality (SAR) is a technology that utilizes projected light to create AR experiences by modifying real objects within a physical environment with perspective-corrected computer generated graphics in real-time and on demand [7,8,9,12,13,14,15,16,17,18]. SAR’s entire system’s FOV corresponds to the user’s natural FOV enabling them to employ their peripheral vision [13,15,19]. The range of the system’s FOV can be extended by adding the number of projectors or using steerable projectors [15,20]. The SAR system supports higher scalable resolution and brightness of generated computer graphics than traditional HMD or handheld display solutions [7,15,18,19]. Pre-warping and color adjustments on virtual data present undistorted video and graphics on arbitrary surfaces in the environment. These techniques counteract reflection and perspective projection effects of the physical objects’ surfaces [7,15]. The accommodation of the eye is easier following the rendering of virtual components near their real-world locations [13,19]. Furthermore, SAR allows lowering of skill level requirement during inspection as it projects digital information onto surfaces [15]. Despite these advantages, SAR is less mature and not well known yet [21].

SAR augments visual virtual objects, for instance texts [7,22,23], three-dimensional computer aided design (3D CAD) objects [23], images, and videos [15], either directly on the target surfaces or indirectly on objects around the target component surfaces [16]. The direct or indirect projection of highly contrasting homogeneously colored components, for example texts and 3D CAD objects, over planar or nonplanar surfaces does not affect legibility performance based on the level of surface texturization [23]. However, their legibility performance is significantly reduced by tactile irregularities on a surface, favoring the label layout technique in [22] that enables reading from arbitrary viewpoints. This approach’s applicability to direct projection of feature-rich virtue objects, for example images and videos, over low contrast projection surfaces is severely limited necessitating an alternate approach. Thus, system developers face a unique challenge during placement across surfaces with miniaturized high-density components, for instance PCBAs. When placement is direct, highly precise yet contrasting augmentation is needed to localize and provide reference to the infinitesimally small component while a wide FOV indicator would occlude several physical parts. On the other hand, when placement is indirect an operator would experience several gaze shifts between the reference and the target location. This limitation in SAR, is yet to be addressed to the best of our knowledge and towards this end, we propose a novel contextualization based on switchable glass. Conceptually, we can automatically control transparency and opaqueness in desired regions as needed. That would imply directly referring to the PCBA through the transparent glass sections as well as contextually placing the reference objects on the opaque regions next to the target location eliminating constant gaze shifts.

Generally, AR has been demonstrated to increase operator’s performance in maintenance [11], assembly, inspection, design and development [13] tasks, as well as an organization’s competitive advantage from efficient training [24]. With regard to inspection tasks using SAR, a number of studies have focused on spot welding [9,13,15,24], 3D objects [25], and none to the best of our ability has focused on safe collaborative/cooperative inspection of PCBAs with contextualization enabled by switchable glass. Therefore, we propose a system that focuses on value addition by leveraging on existing defect information in automatic optical inspection (AOI) to wirelessly localize defects on PCBAs, contextualize rendered reference images on switchable glass and perform gesture-based interactions in a safe manner. This seeks to improve the system’s efficiency and worker’s productivity while enhancing their ergonomic comfort by eliminating constant gaze shifts.

### Related Studies

Projector-based augmentation combines the advantages of natural human interfaces and computer-based user interfaces [19]. These SAR systems allow the tetherless mobility of users without needing any visualization hardware or viewing through a screen [9,15,18,26]. This allows the collaboration of users with the limitation being space availability [9]. Additionally, these systems improve procedural task performance in terms of response time together with number of errors. Furthermore, they have been shown to minimize a user’s cognitive load compared to HMD devices [27]. Such benefits accrue from previous studies, summarized in Table 1, variously applied in gaming, communication, manufacturing, etc.

In gaming, the authors in [12] present RoomAlive, a scalable multi-projector system that enables life-size untethered gaming and can dynamically adapt with its users. This study proposes an automated offline pro-cam calibration based on Gray code patterns to retrieve the pose, focal length and principal components. We base our primary calibration on this technique and open source library available. In communication, a Room2Room [28] life-size co-present telepresence system is presented allowing persons in remote rooms to collaborate. This study seeds our study towards cooperative/collaborative human-machine interaction in manufacturing. In education studies, a SAR system displaying thermal imaging data on real objects is presented in [29]. The system comprises a thermal camera to capture thermal data, depth camera for tracking and registration and a projector that maps the data onto objects. The authors in [27] present a cueing SAR system for predictive target-based and direction-based tasks. The results of their study on two target-based and three direction-based cues indicate that the line cue was most preferred technique in predicting procedural tasks. Target-based cues were most suited to close distances of location of interest while direction-based favored far distances especially while minimizing search of a target location. This study informs our choice on target-based cues for SAR in PCBAs.

In manufacturing maintenance, an SAR system that investigates a seven-task procedure is evaluated in a user study. The system consists of a digital light processing (DLP) projector, industrial camera, markers for tracking, joystick for interaction, and an extra light system for lighting uniformity. Technical information is projected on a motor bike in the maintenance procedure during the study. Their findings show that compared to a paper manual, SAR significantly improves performance, is best suited to complex tasks, better reduces errors and saves completion time [11]. This system, however, is not collaborative and does not contextualize projected reference instructions as we are able to demonstrate. The authors in [30] demonstrate an SAR system that aids manual workers perform assembly tasks by conveying technical instructions in the FOV, monitors the workers’ safety and tracks their postures to eliminate the risk of musculoschelectric seizures. Their proposed system consists of 1 projector, 2 RGB-D cameras and 1 RGB camera. They evaluated their system against light emitting diode (LED) monitor-based display systems where they found SAR systems to be more efficient and minimize the musculoskeletal disorder risk. This study is not cyber-physical and while it benchmarks against LED monitor-based display systems, we benchmark against the OST-HMD visualization system.

In manufacturing inspection, the authors in [15] present a spot-welding SAR automotive inspection system relying on a single projector, computer, an interactive user control pad and markers for tracking. Projected information localizes spot welds, displays corresponding methods of inspection and an operation description sheet. The system can minimize user’s cognitive load, can receive custom vehicle data, and easily accommodates changes in production information. This system, however, suffers from the search for suitable projection surface of highly contrasting virtual components and thus limits the choice of objects that can be projected. Furthermore, at certain angles, 3D aligned projections may have limited visibility requiring the operator to adjust their viewing position.

Furthermore, the authors in [13] present a multi-user collaborative system supported by an optical see-through head-mounted device (OST-HMD) and a pro-cam configuration during a spot welding inspection task. Their system consists of a laser projector-based SAR for global localization and the respective HMDs to provide customized AR instructions to its users. Their system’s effectiveness is evaluated in quality assurance scenario. Our system seeks to be wearable-free thereby not only localizing but also providing customized contextual references on demand via a single setup. Similarly, the authors in [8] demonstrate how the OST-HMD AR glasses can have their FOV extended from 40 to over 100 degrees by integrating a pro-cam hardware, and a ceiling mounted tracking system in FoveAR. Furthermore, a hybrid SAR (HySAR) is presented in [31] as proof of concept combining OST-HMD glasses with an SAR system and is shown to enhance the spatial resolution, intensity, and the dynamic range by 2.7, 1.9, and 2.1 times, respectively. We seek on the other hand to aid operators with an effective SAR inspection system free of wearable computers to enhance their ergonomic comfort.

The authors in [25] present an inspection system that uses a single projector, simultaneously identifying possible defects and displaying its outcome on the component. However, this system suffers from the drawback of unavailable color maps arising from monochromatic augmentation of its video stream which would render it ineffective for our inspection task. On the other hand, our system utilizes all the color streams to display the reference images, thereby reducing the cognitive workload of an inspector. Additionally, in a study by [32], the authors perform onsite rebar inspection of the number of stirrups and their respective spacing in a construction task, saving up to 34% of manual inspection time.

In this study [33], the authors propose a projection based augmented reality assistance for manual component assembly process. The system utilizes Aruco markers for registering the scene, and an RGB camera and a checker board for calibrating the projector-camera setup. They project blinking virtual components directly on the board to localize an assembly region, and assembly parts beside the PCBA. While the posture of the operator is maintained, there are still gaze shifts involved by employing static image references. In this regard, and following [34] where contextual image references are preferred to static ones, we seek to contextualize the reference image by projecting the images right next to the target region of interest within the field of view of the operator.

In regard to studies involving switchable glass, the authors in [35] propose to employ switchable glass for light-based computations as an evolvable hardware. Furthermore, the authors in [18] describe development of a multi-planar volumetric display based on multiple fixed but switchable glass planes. In our case, we have designed and manufactured a single glass plane with each side having 10, 70 mm partitions. Based on various limitations herein presented, ranging from restrictions on color range of displayed content, restrained placement of projected reference objects around the target area necessitating gaze shifts thereby increasing user’s cognitive workload, costly coupling of SAR with wearable visualizers, ergonomic discomfort of operators, duplication of tasks through standalone systems, we hereby present a cooperative/collaborative SAR PCBA safe inspection system cyber physically localizing defects from a remote AOI system and contextually presenting reference images on switchable glass that are interactive via hand gestures. Such an ideal projection surface, according to [19], should be with smooth geometry and possess diffuse light colors. To the best of our knowledge this variation is the first of its kind presented in a formal study.

The authors in [22] present a label layout technique projected directly onto nonplanar and textured surfaces enabling reading from arbitrary viewpoint. This approach is suitable where high contrast between the surface and the projected information is achievable. Especially for static characters or objects of bright and homogeneous color. However, projecting feature-rich dynamically interactive images with nearly same contrast with the projected surface would necessitate an alternate approach, a non-existent limitation in our proposed contextualization based on switchable glass. In a study on the legibility of texts on projected surfaces by [23], the authors observe that legibility performance is not influenced by its texturization but is significantly reduced by tactile irregularities on the surface. Thus for PCBAs, it is anticipated that projected texts would remain legible whether projected directly on its surface or indirectly over the switchable glass. The use of annotations as proposed in [7] reintroduces gaze shifts during PCBA inspection, thereby increasing the cognitive workload of inspectors, a constraint that is minimized/eliminated using the proposed contextualization based on switchable glass.

## 2. Materials and Methods

### 2.1. System Calibration

Towards performing quality inspection [1] notes availability, suitability, functional and properly calibrated inspection tools to be key necessities. However, since projectors have no ability to measure projected image pixels but cameras can [36], we correspond the grey code projected image pixels with known 3D camera locations over uneven surfaces to automatically determine the pose, focal point and principal components of the projector and depth camera using the RoomAlive Toolkit offline [12]. Subsequently, this facilitates the proper registration of virtual components on a 3D scene in global coordinates.

Based on our observation that the estimates provided by Vuforia during PCBA tracking were in a different coordinate frame from the world room coordinates we performed a second local to world calibration. Given that some authors have suggested multiple iterations of the projected pattern until it overlaps a printed pattern, we replace the iterative steps with sliders, providing users with the ability to manually overlap a projected pattern with a PCBA’s centroid. This is performed at four different scene locations after which we compute a homography/transformation matrix enabling us to precisely register virtual components at any 3D position with the desired virtual objects at desirable positions. This process does not need to be repeated for similar board patterns but has to be performed at least once for each unique PCBA board pattern introduced. The method suffers from the subjectivity of human inaccuracy during calibration, and lags during the 3D motion of the PCBA which is time consuming compared to auto-calibration systems. A mitigation to this drawback is that it only needs to be performed once within a setup. The advantage of our calibration approach is irrespective of the target reference object(s) employed (featureless or featured), corresponding detection method(s) generating local coordinate frames, the method can be robustly applied to synchronize each local coordinate frame to the world’s system. Additionally, it supports dynamic projection mapping aligning virtual components with the target object in motion in real-time.

### 2.2. Switchable Glass

Switchable glass or smart glass is an active [37], electrically controllable glass upon its opaqueness or transparency [35]. This glass takes three main forms namely: suspended particle devices (SPD) where the degree of transparency is proportional to applied voltage; electrochromic devices (ECD) that are opaque upon voltage application and the degree similarly vary with applied voltage and the liquid crystal devices or polymer device liquid crystal (PDLC) that only assume either on or off states upon voltage application or withdrawal, respectively. Our study focuses on the latter where the switching time is 100 milliseconds as prescribed by the manufacturer [38] for efficient, real-time, custom, and contextualized projection facilitation. The underlying principle of PDLC is the alignment of liquid crystals upon voltage application allowing light transmission and vice-versa for opaqueness. This is similar to the electrical conductivity of semi-conductors. An ideal projection surface ought to have a smooth geometry and possess diffuse light colors [19] a requirement easily fulfilled by the switchable glass.

Addition of glass in the system introduces possibility of defects commonly affecting optical elements such as breakage, dirt, digs, fingerprints, flaking, cracks, pinholes, rubs, run-ins, scratch, or sleek, stain, bubble or stone [1]. Generally, these defects in the glass are either internal, substrate surface ones, or cosmetic in nature. All these could potentially affect the outcome of the inspection if and when present. Thereby, as a precaution, it is imperative to keep it clean with a scratch free wet cloth and employ dust-free polished caps for scratch-free gesture interaction. Furthermore, the presence of glass requires consideration of the refraction effect upon projected components. This necessitates the use of considerably thin glass substrate and an angle of incidence that eliminates total internal reflection during inspection. We address this issue by specifying a thickness of 11 mm for the switchable glass assembly.

### 2.3. Safe Interaction Methods

The system seeks to provide both target- and direction-based cues for the inspectors without being visually harmful to their eyesight either from high brightness or low resolutions resulting in eye strains. This is attained by projecting highly contrasting reference images and controlling the incident light to the inspection surface. Furthermore, it is imperative that during gesture interaction the hands are safe from a conveyor, herein represented by a planar white surface, or PCBA’s assembly components that tend to have sharp edges. Towards this end, the switchable glass surface does provide that physical barrier for safety while manipulating the projected images.

Furthermore, along with the system continually guiding a user performing an inspection task, it respectively scans his external environment for potential threat to their safety. Particularly, PCBA’s inspection requires a high degree of concentration owing to miniaturized, and dense components which creates an attention deficit to potential threats in their surroundings. Provision of visual feedback during inspection does enhance the workers’ safety in such circumstances. The lifespan of the feedback is as long as the threat is present, and is color coded depending on proximity to the workstation. Color coding allows the inspector to respond accordingly.

Additionally, the proximity of the inspector to the board is of key concern to their safety as well as placement of overhead projector and camera in the setup. Presented with all these factors, we deemed fit the proposed oblique projection system architecture for interactive inspection based on view-independent rendering. Other spatial user interface techniques include employing an additional handheld device as an auxiliary display and interaction surface, body and hand gesture systems, voice interaction, and multimodal techniques. These user interfaces can also be classified as either tangible, physical-virtual tools, or ephemeral [9]. Selection of the spatial user interface should not only be subject to availability but also efficiency and user safety which consequently yields higher throughput for a firm.

### 2.4. Tracking and Registration

Tracking of an object involves determining its 6D pose relative to the camera. Registration, on the other hand, refers to aligning virtual components in situ with the environment to appear at desirable locations as part of the environment. We performed image-based tracking of the actual PCBA by designating it as a marker, thereby rendering the pose determination to be markerless. This allows the motion of PCBA without being physically constrained to the environment, unless it is necessary, and enhances efficiency of required setup time. Image-based tracking by Vuforia is entrenched by rich features available from a PCBA. On the other hand, tracking of the switchable glass is aided by a marker placed across four square cells at the corner of the glass to facilitate origin definition and cell discretization. An image to be contextualized is also primarily centered across 4 square cells. Vuforia-based tracking is available freely to developers, is real-time, and easily configurable with multiple platforms for tracking images and 3D objects in standalone systems or via cloud configuration making it a go for tool, with precise registration offering. Other AR tracking software tools include Wikitude, ARToolkit, EasyAR, etc.

RoomAlive registers virtual components either randomly in a uniform manner, semantically depending on the scene contents determining what to render, constrained to the current location or state of a user, or responsively to user input actions. This advantage is extended to our system by integrating Vuforia-based PCBA tracking to facilitate the real-time alignment of projected virtual components upon transforming the coordinate frames. Additionally, to ensure the projection appearance is invariant to its intended state, we perform geometric and radiometric (photometric) compensation [21] prior to rendering the components upon the scene.

### 2.5. System Architecture and Communication

A key consideration to any SAR system is existence of crosstalk between projected imagery and camera feedback in the visible light spectrum in vision-based pro-cam devices [21]. Additionally, ambient light interference (brightness) [9,11,19,21,39], occlusion [7,19,39], deformation of projected information [7,11], low color fidelity [11], higher latency [11], aliasing artifacts, limited projector’s FOV [11], registration inaccuracy [11,13], projector mounting decision [13], and texture surface complexity (contrast) [11,19,21,39] (rectified by real-time radiometric compensation) causing transparent or confusing visual appearances [11,13,21] are important factors that are not ignorable when designing such a system. The authors in [40] propose a number of algorithm and hardware solutions to these challenges including, but not limited to, dually modulating or reallocating a light source in high dynamic range (HDR) projection, applying a surface modulation technique to project a static HDR image in ambient light, providing natural augmentation by employing high speed and low latency projection systems, subpixel shifting of overlapped multiple image planes for high resolution projection, applying more than three color primaries for multispectral projection, projecting view-dependent images from various directions to optically varying screens for light field projection, etc.

In this regard, our system adopts two Kinect v2 sensors in our setup. This is an RGB-D sensor that synchronizes color (1920 × 1080) and depth (512 × 424) images [41], tracks the PCBA and external marker attached on the glass providing the 6D pose in real-time. Additionally, upon calibration, a second Kinect tracks the human inspector providing the 3D pose joint information of their articulated body in real-time, enabling us to define hand gesture activity by analyzing the temporal patterns in relative joints. A key advantage of this approach is its robustness arising from invariance to viewpoint, light and appearance. Additionally, we employ the retrieved information to calibrate the switchable glass position for subsequent interpolation during human activity recognition. Kinect is a low-cost solution able to that directly provides color and depth information [42] generating 3D position with demonstrated precision [30] rendering it as a practical and accessible solution without interfering with its projection [43].

Furthermore, in our architecture we propose the employment of a single projector. The use of a single projection system is not only cost effective but also negates complexity arising from multi-perspective registration and parallax-based occlusion from multi-projector systems [25]. Since the color and reflectance of the surface are likely to affect projected virtual information [17] we select a diffuse flat surface of the switchable glass as the incident projective image plane. The selected projector for this task has color and light brightness of 2500 lumens and dynamic contrast of 60,000:1 which is sufficiently visible for highly color contrasting virtual objects under ambient conditions. Notably, pro-cam systems are easily deployable by utilizing an off the shelf data projector and camera [18,36].

**Figure 1:**
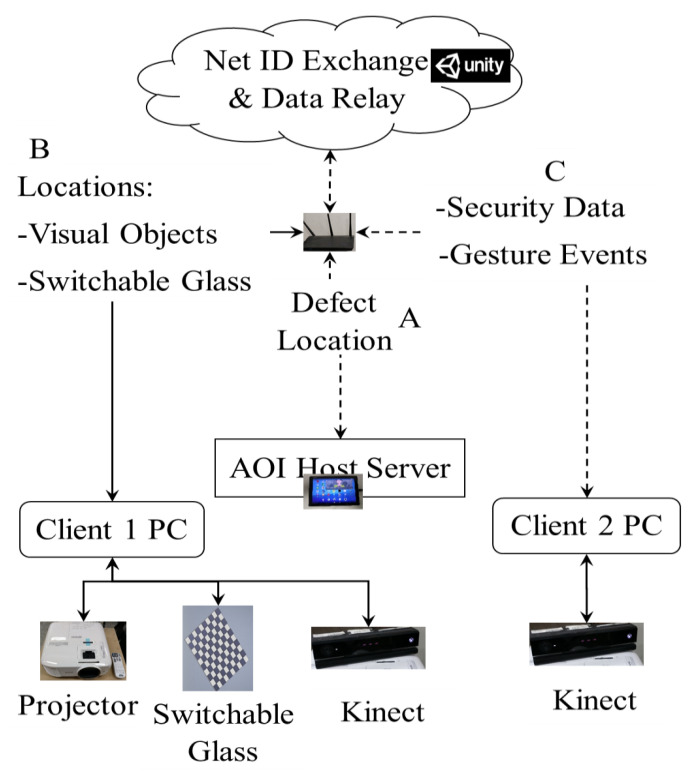
System Architecture.

With regard to communication of PC components within the architecture (Figure 1) to relay information, we adopt wireless communication [44] from the designated server to the clients. The networking infrastructure is developed in Unity3D, a cross-platform development platform that supports wireless relay of messages across the transport layer based on user datagram protocol (UDP). In the present setup, the AOI defect location information (A) is wirelessly transmitted by user requests received through a Tablet PC to all clients. This information localizes the defect area on a PCBA and the contextualized reference image. The target defect area is visualized via a highly color contrasting animated rectangular bracket aided by the projector and the PCBA tracked by a Kinect v2 sensor. Furthermore, the reference image is contextualized assisted by a switchable glass. The generated virtual component locations (B) are communicated to other clients in the system in real-time to facilitate initial gesture events calibration and subsequent generation. While tracking the gestures of the inspector, the second Kinect v2 sensor also scans and monitors the environment for potential threats, consequently generating security events (C) communicated to other clients in the system for visualization by the inspector. The Unity3D web service integrates all this communication in real-time for a synchronized and seamless inspection process via Wi-Fi following IEEE 802.11 b/g/n protocol [45].

### 2.6. System Design and Implementation

**Figure 2:**
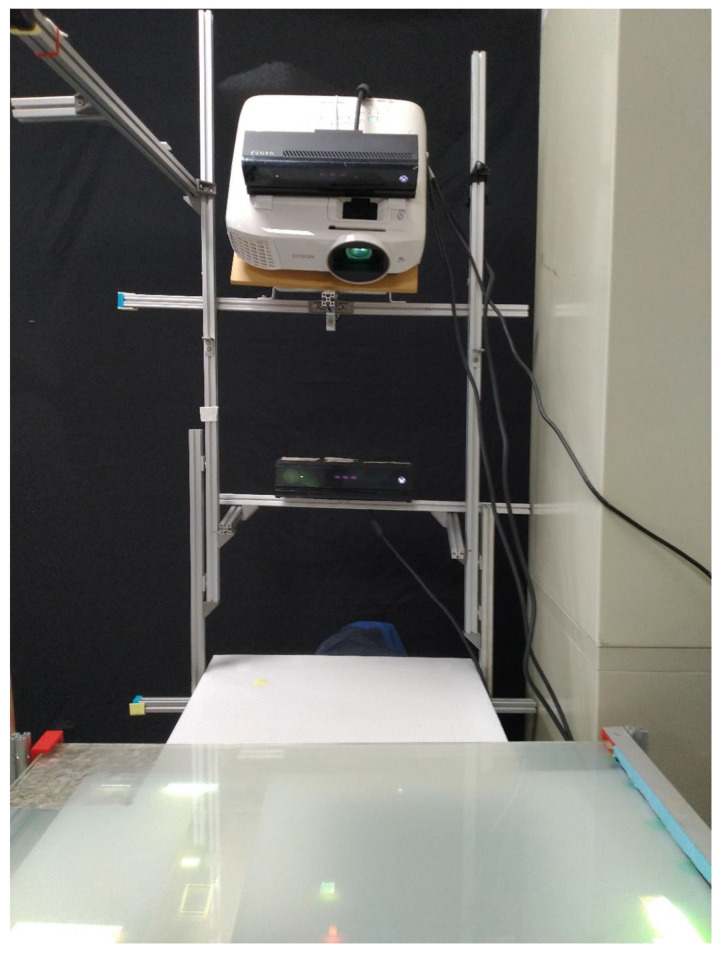
Developed Inspection System Setup.

In this section, we detail the proposed setup’s (Figure 2) requirements including calibration, interaction, contextualization, safety and evaluation in a user study.

#### 2.6.1. Safely Interactive Inspection Task

**Figure 3:**
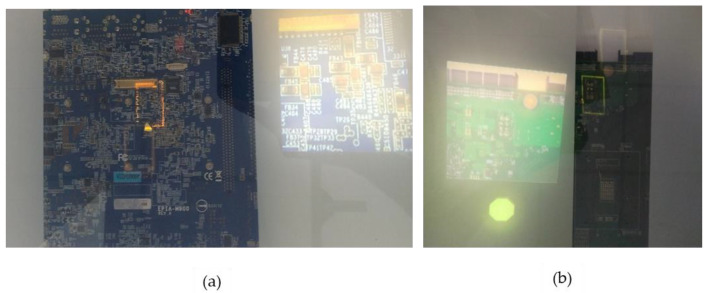
(**a**) Contextualized Reference Image Small Sized PCBA; (**b**) Contextualized Reference Image Medium Sized PCBA.

The task involves validating the presence or absence of a remotely localized defect on a PCBA by a user in the safest and most efficient way utilizing SAR. This involves contextualizing an interactive reference image enabled by an integrated switchable glass and highlighting the target defect region using a highly color contrasting rectangular bracket as shown in Figure 3a,b. The safety information of the inspector is to be communicated in real-time for user timely response and without being disruptive to the inspection process. Furthermore, for a large PCBA, the inspector is provided with arrows overlaid with exact distance text, especially to compensate for regions outside the projector’s field of view. The colored blinking rectangular bracket together with exact distance text are target-based cues that efficiently guide a user to a specific location. On the other hand, the arrow along with partial occlusion provided by the switchable glass are direction-based cues that orient a user to the general location [27]. Generally, target and direction-based cues are respectively applied to close and far distances, respectively.

#### 2.6.2. Inspection Items and Method

**Figure 4:**
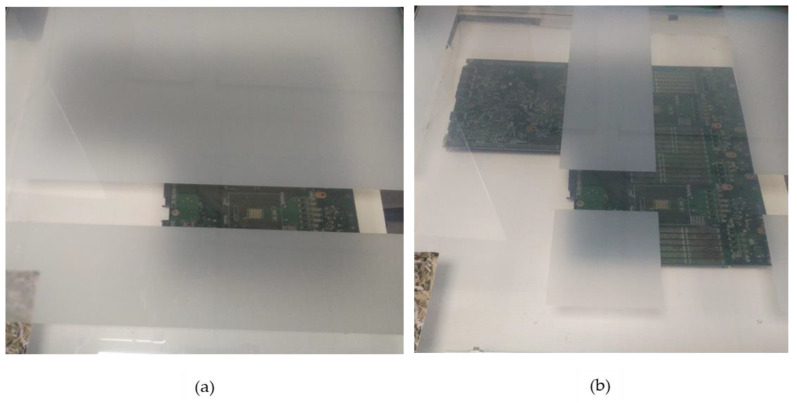
(**a**) Full Occlusion; (**b**) Partial Occlusion.

Small, medium and large PCBAs are to be inspected for the absence/presence of defects remotely localized by an AOI system. A prescribed defect location is to be directly highlighted over the PCBA by a blinking rectangular bracket. Blinking targets of high color contrast are selected given that inspectors are likely to gaze earlier and for long durations at anomalous objects in a scene than congruent ones. Furthermore, a corresponding reference image is to be contextualized aided by switchable glass to eliminate/minimize gaze shifting. The contextualization is to be performed via a fully occluded PCBA, revealing the desirable target location as illustrated in Figure 4a or a partially occluded PCBA exposing most of the PCBA as shown in Figure 4b. The effect of the resulting crosstalk will be analyzed on the users participating in this study. On the other hand, a static reference will be provided during the study to evaluate the resulting efficiency from contextualization.

#### 2.6.3. Calibration Implementation

The integration of various modules necessitates the calibration of the entire system. Specifically, in our study, this involves three areas:

1. Projector camera (Pro-cam) calibration to provide the pose, focal length and principal point of the projector camera configuration in world coordinate frame.
2. Local coordinate frame determined via Vuforia’s transformation to the system’s world coordinate frame.
3. Local glass coordinate frame transformation to the second Kinect’s coordinate’s frame and the system’s world coordinate frame.

**Figure 5:**
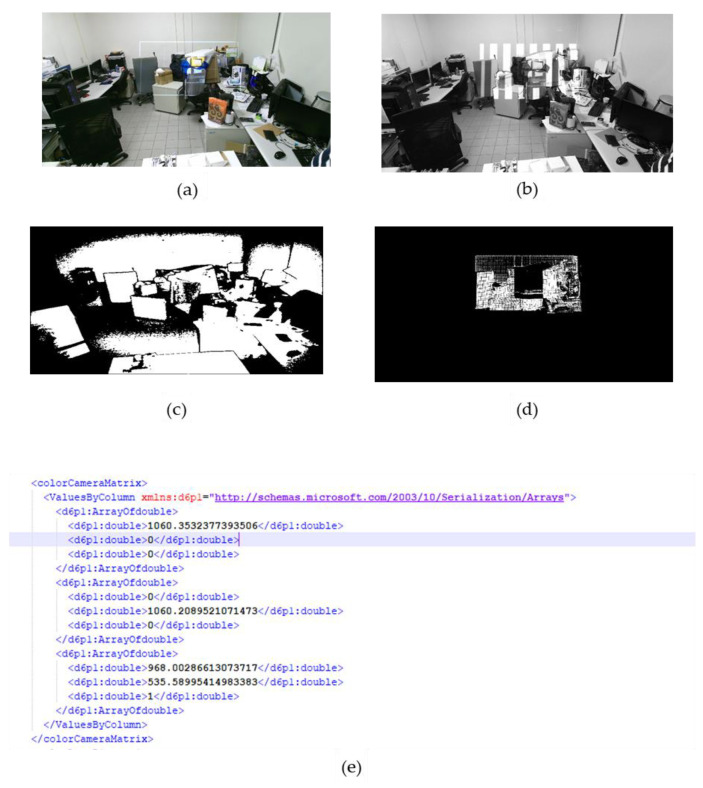
(**a**) Irregular Projection Surface. (**b**) Projected Gray Code Patterns on Irregular Surface (**c**) Camera Mask of Irregular Surface (**d**) Projector’s Valid Mask of Irregular Surface (**e**) Color Camera Matrix Excerpt.

The pro-cam calibration is undertaken to determine the intrinsic and extrinsic properties of the projector and Kinect using structured light aided by RoomAlive toolkit. The principal point, focal distance and distortion coefficients are retrieved through a DLT algorithm on the projected structured light on irregular surfaces by corresponding projector pixels with known 3D physical locations as indicated in Figure 5. Secondly, to determine the transformation required from Vuforia’s local coordinate frame to the system’s frame, we project a predetermined object in the local frame to our panel and manipulate its position to its desired position using projected sliders and record the corresponding positions. This is iterated at four locations to the PCBA, thereby working out a transformation that maps between the two frames as follows:

(1) $$T=OI^{-1}$$

where: O are coordinates of four points in world coordinates, while I are corresponding points in Vuforia’s coordinate’s frame. This transformation is worked out initially and the values stored in a text file for subsequent utilization. Based on this calibration, the registration precision of the target defect position with and without the glass is provided in table. The precision is similar for contextual reference image with the switchable glass present. Notably, we do not need to recalibrate the system with the glass present, as the error introduced due to refraction is insignificant and in all cases the target defect position is highlighted by the blinking bracket.

**Figure 6:**
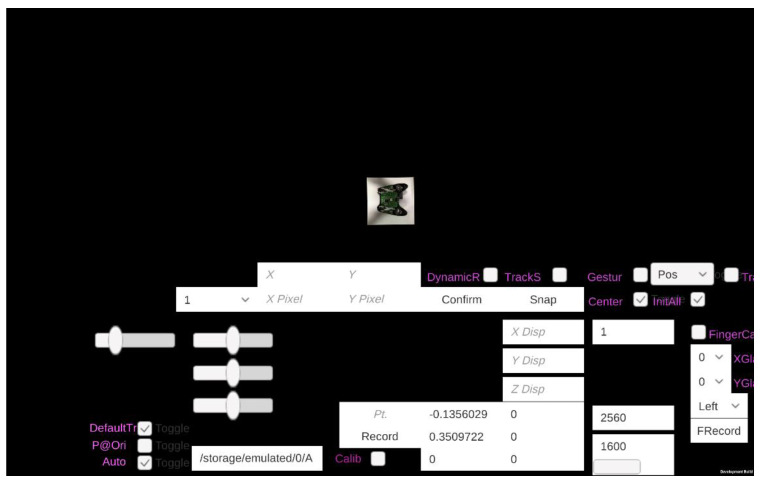
Custom User Interface.

The third calibration involves determining the position of the glass relative to the second Kinect. This is determined by recording the 3D positions of each hand at the corners relative to the Kinect for the subsequent interpolation for a random intermediate glass position. We provide a custom user interface (UI) (Figure 6) that guides a user by simple button selections while recording and subsequently reusing the calibration values. Its position relative to the first Kinect follows the same transformation T determined in the second step aided by a marker placed across four square cells at the corner of the panel.

#### 2.6.4. Switchable Glass Configuration

**Figure 7:**
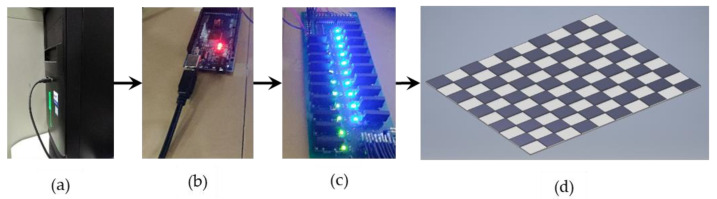
(**a**) PC that serially communicates with Arduino; (**b**) Arduino Mega Microcontroller; (**c**) Custom PCBA interfacing the Switchable Glass; (**d**) 3D CAD design of the Switchable Glass.

The PDLC designed in our study is a 700 mm × 700 mm plate divided into 10 sections across its length and width. This implies that 100 cells are independently controllable, enabling our system to have a variety of configurations as needed. Since only two states are possible, we select the AQZ202 relays for electronic switching. The solid state relays (SSR) are chosen for their fast performance, enhancing the real-time characteristics of the system, eliminating of the need for a counter electromotive protection diode on the input side while maintaining quiet performance compared to the noisy reed and mechanical relays. Additionally, we interface the relays to the Arduino Mega microcontroller with the aid of Darlington transistors which aid us further in optimizing the space utilized. Subsequently, the custom board communicates with the system via serial communication through commands remotely relayed to selectively form opaque/transparent regions as desired, enabling contextualization of reference images. The opaqueness or transparency arising thereof is from the switching on or off of a 48 V supply to the glass plane. The resulting configuration is a shown in Figure 7.

Tracking of the switchable glass is aided by a marker placed across four square cells at the corner of the glass to facilitate origin definition and cell discretization. An image to be contextualized is also primarily centered across four square cells.

#### 2.6.5. Reference Image Contextualization

A target reference image is contextualized on the switchable glass by determining the midpoint of four cells closest to, and without overlapping, the target defect’s region of interest. This follows a relation [46,47] of the form:

(2) f(x)={⌊(x−δ)/d⌋d−d,0≤x<0.5(n−1)d⌈(x+δ)/d⌉d+d,0.5(n−1)d≤x<ndf(y)=0∀yf(z)=z/dd+d∀z

where d is the discretization magnitude for a cell, n is the total number of cells along a length or width, δ is infinitesimally small coefficient, floor(x) = ⌊x⌋, ceil(x) = ⌈x⌉ [47] and round(x) = x [46]. The coefficients of x, y and z are relative to the marker on the switchable glass. Contextualization of a reference image within an inspector’s field of view eliminates constant gaze shifts during inspection, enhancing the efficiency and precision of the process, as there is minimal or no cognitive workload. Generally, reference image contextualization across four cells of the switchable glass follows an algorithm of the form shown in Table 2, with the corresponding switchable glass cells selected employing equation III:

(3) $$\begin{array}{c} f(x)=n-\left|focalILPos.z\right|*d^{-1}\\ f(y)=0\\ f(z)=n-\left\lceil focalBoxLPos.x\right\rceil *d^{-1} \end{array}$$

#### 2.6.6. Safety in Human Machine Interaction/Safe Spatial User Interface

**Figure 8:**
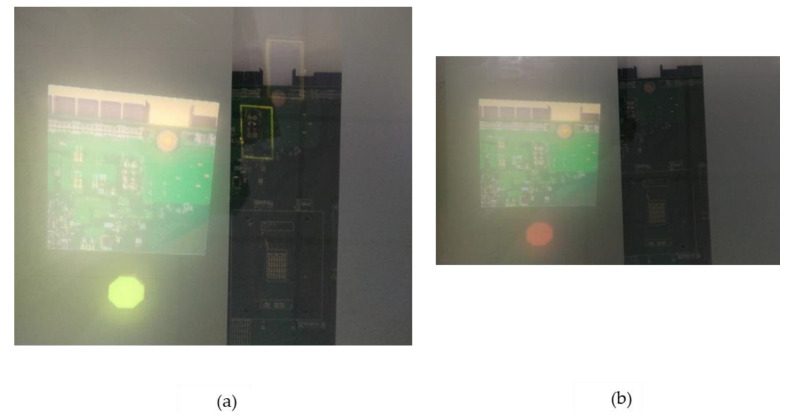
(**a**) Yellow disc appended to reference image. (**b**) Red security disc appended to an image.

**Figure 9:**
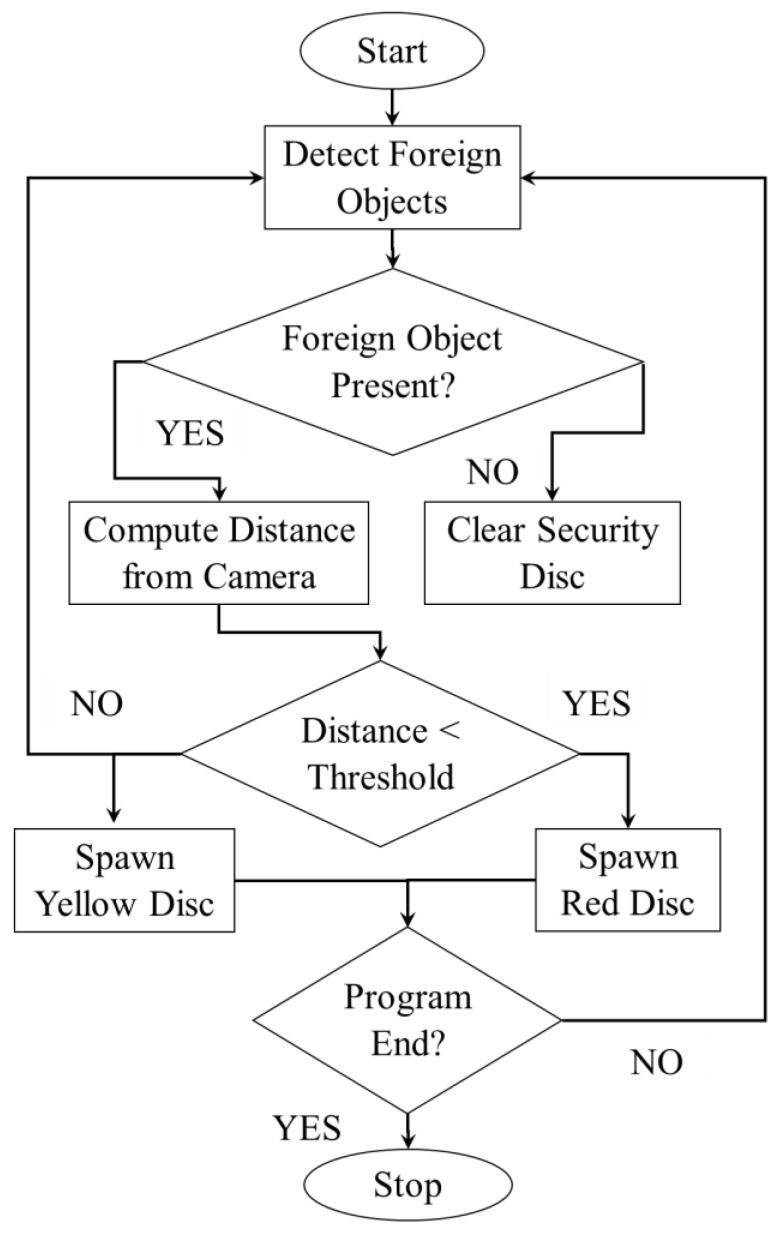
Flowchart algorithm of Safe Human–Machine Interaction.

We have a second Kinect 2.0 in our setup responsible for twofold functions: monitoring the security state of the inspection area by detecting the presence of humans/robots in the scene and allowing gesture interaction of displayed items. Upon detection of a human/robot, a colored security disc is displayed appended to the spawned reference image as demonstrated in Figure 8a,b. This ensures real-time notification to the inspector without departing from his field of view, especially where the inspector needs to respond. The color of the security disc depends on the distance of the foreign object/person in the buffered inspection area. A yellow disc implies no response is required while a red one implies high caution should be observed. Figure 9 details the monitoring algorithm employed by the second Kinect.

#### 2.6.7. Hand Gesture Interaction

We performed three hand gestures to interact with the contextually rendered reference images on the switchable glass executing three functions as follows:

1. Instant predetermined magnification to the rendered image.
2. Refined zoom in or zoom out of the rendered image.
3. Accessing previous defect position already examined.

**Figure 10:**
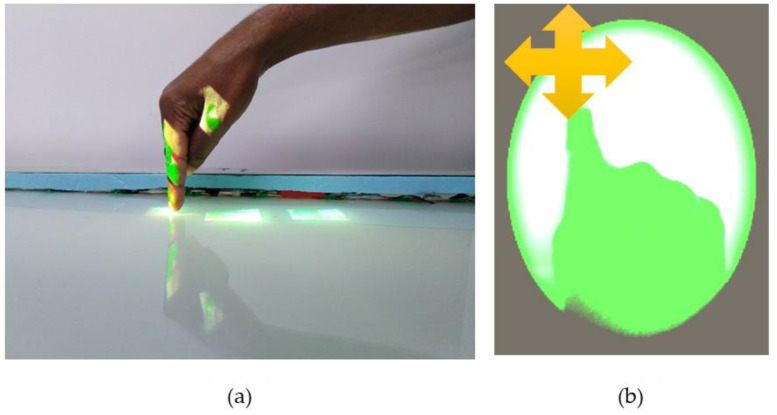
(**a**) Gesture Initialization; (**b**) Gesture Initialization Icons.

Instant/coarse magnification to the rendered image is performed using the right index finger, refined zoom in or zoom out is performed using the right and left index fingers, while access to the previous position is undertaken using the left fist arm. Prior to executing any of the functions, we perform initialization on the glass with the respective hand required to interact with the image as indicated in Figure 10a. Initialization is required to eliminate unwarranted events that are unintentionally generated. We provide an image of the hand to be utilized for each task as well as CAD representation of the respective task for intuitive visualization as indicated in Figure 10b. Additionally, we vary the color of the CAD objects for the corresponding task initialized to provide visual feedback to the inspector. Finally, exiting any of the states is achieved by successfully executing the function or by raising the respective arm above a certain threshold. Notably, the refined zoom in/zoom out is performed within 7 s before exiting the function.

#### 2.6.8. User Study

A user study is to be performed to evaluate the effectiveness of the developed system across three PCBAs. Specifically, this study will address the impact of levels of occlusion on an inspection task, compare this system with the OST-HMD-based inspection system, and evaluate contextually against static display modes in a projector-based inspection. Consequently, the null hypotheses adopted in this study are: There is no difference in usability of projectors to OST-HMD devices during inspection. Full and partial occlusion cause no significant differences towards an inspection task. Contextual and static mode of referencing have no significant impact towards inspection.

### 2.7. Experimental Hardware and Software Setup

**Figure 11:**
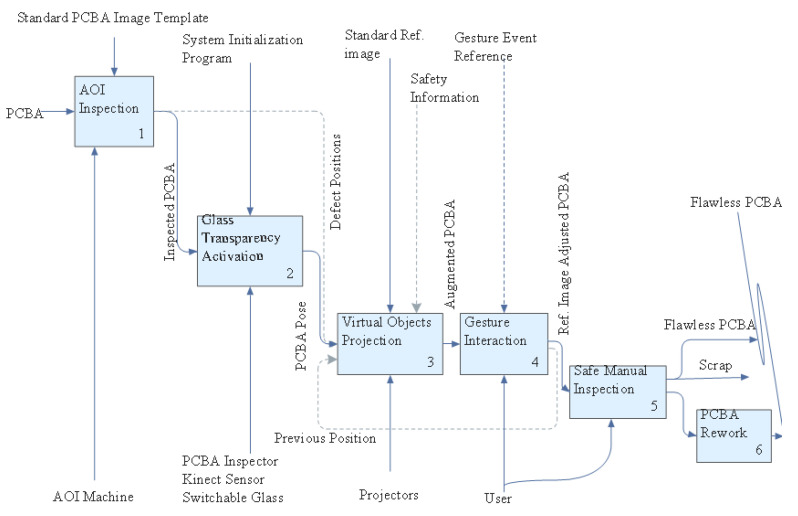
Implemented System Flowchart.

Our current implementation, as demonstrated in Figure 11, is a client–server network architecture type where a Sony tablet UI is designated as the AOI host’s interface for generating commands for two remote client PCs. A desktop PC (16 GB RAM Memory, 3.6 GHz Intel Core i7-7700, 4 Cores, 8 Logical Processors, Intel HD Graphics 630 1GB RAM Graphics card, NVIDIA GeForce GTX 1050 2GB RAM, sourced in Taipei, Taiwan) and a Hewlet Packard laptop (8GB RAM Memory, 2.4 GHz Intel Core i7-5500U, NVIDIA GeForce 940M, sourced in Taipei, Taiwan) are the two configured remote clients. A mouse is employed during calibration of the PCBAs for slider motion in the desktop PC for fast input. We use a 3LCD Epson projector with a native resolution of 1980 × 1020, 2500 color and brightness lumens and a contrast ratio of 60,000:1 (online source, USA). Finally, two Microsoft Kinect for Windows v2 sensors are utilized in this setup.

The main application was developed using Unity3D 2018.2.0b7 (64-bit) in C# programming language, laying out the logic, integrating Vuforia for markerless 6D pose estimation, and networking the AOI server with the pro-cam client. Vuforia software was employed for the 6D markerless tracking of the PCBA board.

## 3. Results

### 3.1. Study Design

In total, 30 respondents drawn across different departments in the graduate school of the National Taiwan University of Science and Technology evaluated the developed inspection system both physically and via a structured questionnaire. Subsequently, this would facilitate the derivation of reliable conclusions on the study. The questionnaire was structured with both closed and open-ended questions, with a 7-point Likert scale being appropriately adopted. Given that Likert scales are treatable as interval data, this supports reliance on parametric test result analysis [28]. Their mean age was 28.5 years with 66.7% being from department of mechanical engineering, 20% equally from civil and construction engineering together with chemical engineering, 6.7% with an industrial management background, and the remainder from electrical and computer science engineering backgrounds. Notably, all respondents were male, with 46.7% having prior experience with AR and 33.3% having prior experience maintaining PCBAs. Results from the user study were analyzed using SPSS v.20. Testing of the system was performed following the modes of display in Table 3 on the projector-based inspection, while for the OST-HMD the medium PCBA was employed with contextualized images for each respondent.

We examined the preference of respondents’ suitability in conducting inspection across three PCBA sizes. Results indicate a preference to inspect smaller PCBA’s over medium and large PCBA’s with means of 5.43 (σ = 1.675), 5.17 (σ = 1.020) and 4.87 (σ = 1.717), respectively. This was attributable to the ease and speed of inspection based on required mobility, reference image resolution and PCBA component composition. However, the difference between means was not statistically significant (*p* = 0.054, *p* = 0.81 and *p* = 0.563, respectively) based on an analysis of variance (ANOVA) on the given reasons at 5% level of significance. A paired difference in means was significant for the rated variations between the small board and the medium one together with the small versus large board (*p* = 0.038 and *p* = 0.049, respectively). However, there was no significant difference in preference between the medium sized and large board (*p* = 0.184). Furthermore, the calculated absolute registration error for the blinking rectangular bracket were obtained as shown in Table 4. This demonstrates that the effect of refraction to existing absolute error from calibrations is random and not significantly different. Therefore, introduction of the glass did not require the system to be recalibrated with regard to its registration.

### 3.2. Projector Based AR Vs OST-HMD AR

**Figure 12:**
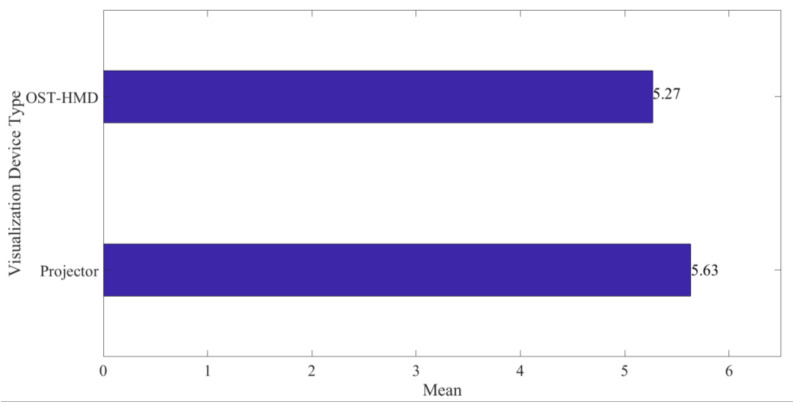
Visualization Device Type against Average Usefulness.

A comparison between projection-based PCBA inspection and OST-HMD inspection was performed. Results indicate the projection-based system to be more suitable for PCBA inspection compared to OST-HMD, with means of 5.63 (σ = 1.245) and 5.27 (σ = 1.285), respectively, as presented in Figure 12. This was largely attributable to the ergonomic convenience to the inspectors, the interactivity of the system, and the quality of displayed image. Other reasons were differences in field of view, ability to collaborate, and noise from partial visibility of the board in the OST-HMD. However, the difference between two means was not significant at a 5% level of significance. This was attributable to the short duration the experiment which took approximately 25 min on average per respondent. Therefore, for infrequent, short duration-based PCBA inspection, the OST-HMD would also be a suitable alternative to perform a double-check inspection task.

The respondents further assessed the usefulness of projector-based AR with switchable glass enabled contextualization in executing various functions of inspection. Search attribute was the most aided, followed by access, initiate, decision and rework progressively with means ranging from 5.90 to 4.77.

### 3.3. Occlusion Impact on Efficiency

**Figure 13:**
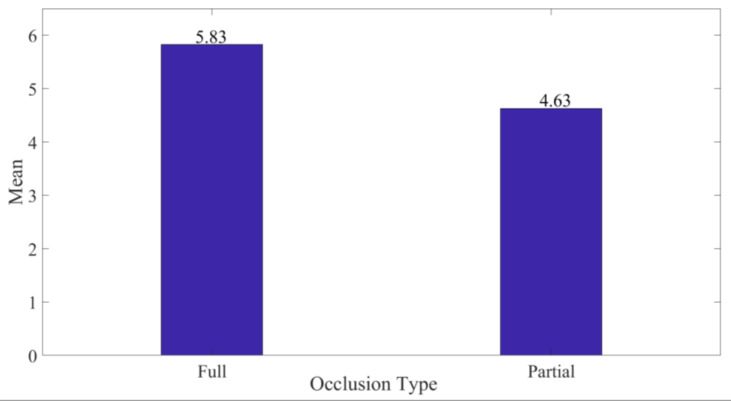
Average Utility against Occlusion Type.

With regard to occlusion, the full level of occlusion was best rated averaging 5.83 (σ = 1.289) compared to partial occlusion at 4.63 (σ = 1.65) on the 7-point Likert scale as indicated in Figure 13. This was mainly attributable to the increased focus provided to users during inspection. Additionally, the mean variation was significant (*p* = 0.012) at a 5% level of significance. Furthermore, a comparison of mode 3 and 5 in Table 2 for medium and large PCBAs, respectively, assessed under similar conditions indicates that inspection under full occlusion took an average of 9.4 s for all respondents against an average of 10.7 s under partial occlusion.

### 3.4. Occlusion Impact on Efficiency

**Figure 14:**
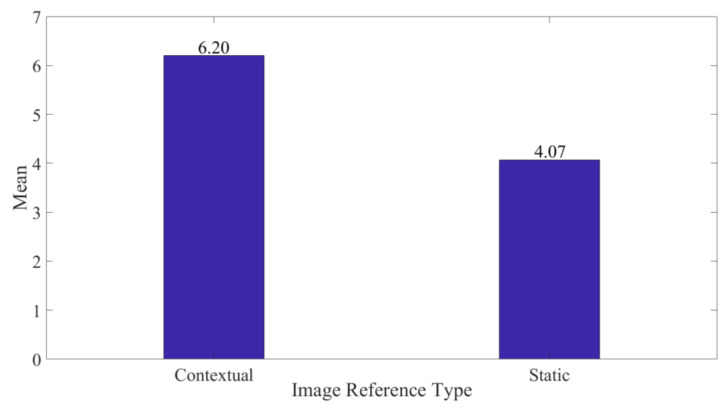
Average Preference against Image Reference Type.

Furthermore, we analyzed preference for utilizing static against contextual reference images during inspection. The results indicate greater preference to contextual images averaging 6.20 (σ = 1.157) against 4.07 (σ = 1.874) of static ones as demonstrated in Figure 14. This was attributable to fewer gaze shifts, ease, and intuitiveness of referencing during inspection. Subsequently, the variation was found to be significant (*p* = 0.001) at a 5% level of significance. This implies that the contribution of the switchable glass towards the inspection efficiency is high.

### 3.5. Spatial Interaction Methods

**Figure 15:**
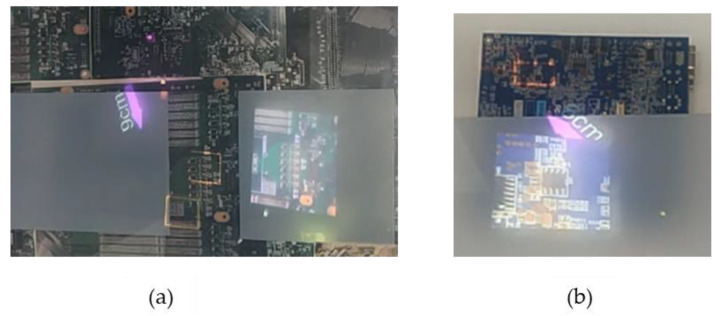
(**a**) Arrow with overlaid distance text on large PCBA. (**b**) Arrow with overlaid distance text on small PCBA.

The target defect region of interest, highlighted by a blinking rectangular bracket (Figure 8a) every 300 milliseconds, is localized by scaling the input pixel positions to the initial image template resolution of the PCBA. The blinking rectangular bracket employed across each board had the physical dimensions shown in Table 5. On the other hand, the arrow overlaid with distance text (Figure 15) was spawned for 5 s to reorient users over the large PCBA given that some areas were outside the FOV of the projector. Equally, it was overlaid over the medium and small PCBAs for evaluation of its effectiveness during the user study. The overlaid distance was calculated from the arrow tip to the midpoint of the defective region of interest.

**Figure 16:**
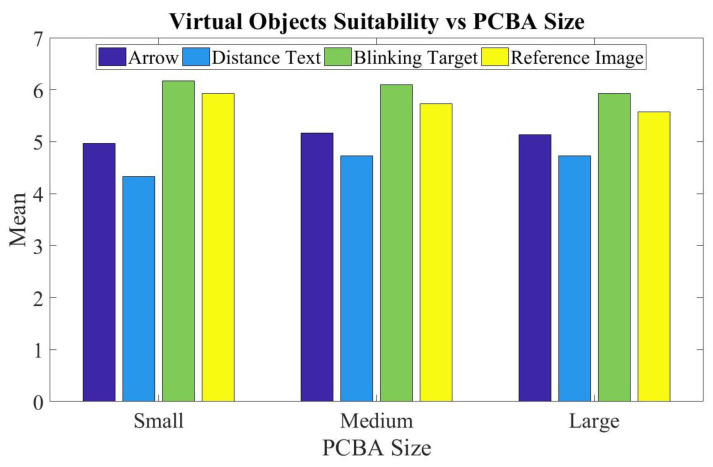
Virtual Objects Suitability versus PCBA Size.

To assess the usefulness of displayed virtual objects during inspection, the respondents visually interacted with blinking rectangular brackets, reference images, and arrows overlaid with distance text. The results in Figure 16 indicate the localizing blinking bracket and reference images to be most suitable, with distance text being least suitable across the three PCBA sizes. This is attributable to the efficiency arising from having defect localization information visually available to users in a wide field of view and orientation provided through visual shifting of the switchable glass cells.

With regard to the preferable interaction mode during visual inspection of PCBAs, tablet interaction was rated higher than the hand gesture input mode, with means of 5.43 (σ = 1.194) and 4.40 (σ = 1.50), respectively. This was attributable to the stability of the tablet input mode compared to the hand gestures. In total, 16.7% noted that hand gestures were more convenient to the tablet mode and with further development and stability would significantly prefer hand gestures. The difference between the two means was found to be significant (*p* = 0.004) at a 5% level of significance. Furthermore, the zoom and previous/next command were better preferred using the tablet compared to the hand gestures.

## 4. Discussion

The results obtained from the study indicate usability of the system with regard to contextualization of reference images during an inspection task. This is attributable to reduced/elimination of gaze shifts during an inspection task that consequently leads to increased throughput. The results are similar to those of [48], who compared AR-based annotations over handheld tablet interfaces against static pictures. Furthermore, projector-based inspection with switchable glass contextualization is preferred to OST-HMD based PCBA inspection. This was a result of the ergonomic convenience during inspection, enhanced field of view, minimal crosstalk from the board while taking a reference and ability to collaborate. This result confirms the finding from [23] that projected that AR can be used effectively for displaying technical information with enhanced safety, ergonomics and autonomy over OST-HMD [49]. In the short-run, the difference was not significant, as the impact of wearables towards comfort was mitigated by the reduced experiment time. Additionally, the study found that full occlusion was significantly preferred to partial occlusion, owing to the enhanced focus during inspection. With regard to the appropriateness of the interaction tools, animated rectangular bracket and reference images for localizing defects coupled with orientation capability of the switchable glass offers unmatched reliable inspection tools. Similarly, the study in [27] shows a higher preference on blinking targets to arrows towards reducing the mental effort of a user with simultaneous faster response time. Furthermore, owing to the stability of the input method, the tablet input mode was most preferred while interacting with the system. Additionally, inspecting smaller sized PCBAs was preferred over medium and large PCBAs. This could be attributable to the easier and faster inspection based on minimized inspector mobility over the PCBA surface. With regard to the effect of refraction on the absolute registration error, the results demonstrate the impact to be random without significant difference from registration performance in absence of the glass. Generally, the absolute registration error increases with the increase in PCBA size.

## 5. Conclusions

This study presents a novel, wirelessly collaborative system leveraging on existing defect information from an AOI system for safe, efficient manual PCBA inspection with reference information contextualized using a custom designed and manufactured switchable glass. The system demonstrates its advantages, robustness and uniqueness by being evaluated against three PCBAs of small, medium and large sizes. Specifically, it outperforms a wearable state of the art system during a remote PCBA inspection task owing to its unlimited field of view, comfort and convenience to users, and its simple intuitive learning experience to new users. The development further shows an effective calibration method that seamlessly assimilates with existing SAR systems without the need for additional modification to existing transformations. Its ability to provide targeted occlusion, contextualization and fast reorientation to a user synergizes not only their efficiency but also enhances safety during interaction.

In future, based on the user study’s recommendations, we aim at increasing the resolution of the reference images provided, stabilize the hand gesture interaction, match precisely the highlighted defect region with an exact image for reference, integrate text instructions during gesture interaction, and provide additional radiometric compensation to distorted images.

## Figures and Tables

**Table 1:** Summary Related Studies.

Title			HySAR [31]	SAR Cues [27]	Automotive [11]	Phone [30]	PCBA [33]	Automotive [15]	Automotive [13]	Laser SAR [24]	Large Scale [9]	3D Object [25]	Proposed
**Manufacturing Task**	Inspection	Spot Welding						×	×	×	×		
		3D Objects										×	
		PCBA											×
	Maintenance									×			
	Assembly	Automotive Engine			×								
		PCBA					×						
		Mobile Phones				×							
	Industrial Design										×		
**Augmentation**	Text				×	×	×	×	×		×	×	×
	Images						×		×			×	×
	3D CAD		×	×	×	×	×	×	×	×	×	×	×
**System Components**	Projectors		1	1	1	1	1	1	1	1		1	1
	Camera		1	1	1	2	1	1	1	1		1	2
	HMD		1						2	1			
	Handheld												1
	Device Mobility		No	No	No	No	No	Yes	No	Yes	No	No	No
**Controller**	Physical			×	×		×	×			×		×
	Physical-Virtual										×		
	Ephemeral		×								×		×
**Collaborative**	Human–Human								×				
	Human–Machine												×
**Tracking**	Markers		×		×		×	×		×			×
	Markerless			×		×			×	×	×		×
**Visualization**	Switchable Glass												×
	PC Monitor									×			
	HMD		×						×				
	Object Surface		×	×	×	×			×	×		×	×
	Object Periphery				×	×	×		×			×	
**Interaction**	Gestures												×
	Controller/Mouse			×	×			×			×		×
	Tablet												×
**Safety**	External Risk												×
	Posture risk					×							

**Table 2:** Reference Image Contextualization Algorithm.

xPixel: x pixel value defect position
yPixel: y pixel value defect position
xResolution: Width Resolution of the reference image
yResolution: Height resolution of the reference image
refImageGPos: position of the reference image relative to the world coordinate frame
refImageGPCBAPos: position of the reference image relative to the PCBA coordinate frame
refImageLPos: position of the reference image scaled from 0 to 1 relative to the PCBA
rectBoxLPos: position of the rectangular box scaled from 0 to 1 relative to the PCBA
rectBoxGPCBAPos: position of the rectangular box relative to the PCBA coordinate frame
sGlassLabels: Absolute reference image and rectangular positions for switching on and off the glass cells
**Input:** xPixel, yPixel, xResolution, yResolution
**OutPut:** refImageGPos, refImageGPCBAPos, refImageLpos, rectBoxPos, sGlassLabels
rectBoxLPos←scale xPixel & yPixel to xResolution & yResolution, respectively
rectBoxGPCBAPos←LTrans * rectBoxLPos
rectBoxGPCBAPosSG←rectBoxGPCBAPos
rectBoxLPosSG←retrieve from rectBoxGPCBAPosSG
**If** (Contextual) **then**
refImageLPos←f(x, y, z) in equation **2**
**Else**
refImageLPos←f(x, y, z)Static
**End if**
refImageGPCBAPos←retrieve from refImageLPos
refImageGPos←GTrans * refImageGPCBAPos
sGlassBoxLabels←f(x, y, z) in equation **3**
**If** (full occlusion) **then**
Switch off all cells except the column with the defect←f(sGlassBoxLabels)
**Else**
Switch on all cells except 2 rows in the nearest neighbor around rectangular box←f(sGlassBoxLabels)
**End if**
**return**

**Table 3:** Projector-based configuration tests.

Modes	PCBA Size	Reference Image		PCBA Occlusion	
		Contextual	Static	Full	Partial
1	Small	×			×
2	Medium	×		×	
3		×			×
4			×	×	
5	Large	×		×	
6		×			×
7			×	×	

**Table 4:** Absolute Registration Error

PCBA	Dimensions (mm)	Absolute Registration Error (mm)	
	Size (L × W)	Glass Absent	Glass Present
Small	170 × 170	2.940	3.186
Medium	495 × 423	4.165	3.599
Large	760 × 435	3.872	3.934

**Table 5:** Rectangular Bracket and Arrow Dimensions.

		Rectangular Bracket (mm)		Arrow (mm)	
PCBA	Size (mm)	Length	Width	Length	Width
**Small**	170 × 170	33.1	21.1	83.0	11.2
**Medium**	495 × 423	45.1	25.2	38.2	7.4
**Large**	760 × 435	41.9	35.1	58.2	5.8
